# Sindbis virus outbreak and evidence for geographical expansion in Finland, 2021

**DOI:** 10.2807/1560-7917.ES.2022.27.31.2200580

**Published:** 2022-08-04

**Authors:** Maija T Suvanto, Ruut Uusitalo, Eveline Otte im Kampe, Tytti Vuorinen, Satu Kurkela, Olli Vapalahti, Timothée Dub, Eili Huhtamo, Essi M Korhonen

**Affiliations:** 1 Department of Virology, University of Helsinki, Helsinki, Finland; 2 Department of Veterinary Biosciences, University of Helsinki, Helsinki, Finland; 3 Department of Geosciences and Geography, University of Helsinki, Helsinki, Finland; 4 Department of Health Security, Finnish Institute for Health and Welfare, Helsinki, Finland; 5 ECDC Fellowship Programme, Field Epidemiology path (EPIET), European Centre for Disease Prevention and Control (ECDC), Solna, Sweden; 6 Institute of Biomedicine, University of Turku, Turku, Finland; 7 Department of Clinical Microbiology, Turku University Hospital, Turku, Finland; 8 HUS Diagnostic Center, HUSLAB, Clinical Microbiology, University of Helsinki and Helsinki University Hospital, Helsinki, Finland

**Keywords:** Sindbis virus, Pogosta disease, Finland, outbreak, mosquito-borne virus

## Abstract

Sindbis virus (SINV) caused a large outbreak in Finland in 2021 with 566 laboratory-confirmed human cases and a notable geographical expansion. Compared with the last large outbreak in 2002, incidence was higher in several hospital districts but lower in traditionally endemic locations in eastern parts of the country. A high incidence is also expected in 2022. Awareness of SINV should be raised in Finland to increase recognition of the disease and prevent transmission through the promotion of control measures.

Sindbis virus (SINV) (*Togaviridae* family, *Alphavirus* genus) is the causative agent of Pogosta disease, a typically self-limited disease with common symptoms of rash, arthralgia, myalgia and fever [1,2]. In some cases, arthralgia and myalgia can persist from months to years and negatively affect quality of life [2,3]. While circulation of SINV has been reported in mosquitoes and birds globally, symptomatic human infection has almost exclusively been reported in Finland, Sweden, Russia and South Africa [4]. However, larger outbreaks and annual cases are reported only from Finland, where the SINV seroprevalence in the general population was 5.2% in the years 1999 to 2003 [5].

Laboratory diagnosis of Pogosta disease is done using ELISA, and paired samples are often needed because the antibody response against SINV develops slowly [6]. In Finland, SINV has been endemic since the 1960s, and the first epidemic occurred in 1974 [1,7]. A laboratory-confirmed case is defined as either detection of SINV IgM and IgG in a single serum specimen or seroconversion between paired specimens. The laboratory-confirmed cases have been notified to the National Infectious Diseases Register (NIDR) since its implementation in 1995 [8]. A total of 566 laboratory-confirmed cases were notified in 2021, compared with an average of 158 annual cases between 1995 and 2021, making it a notable outbreak year. Similarly, high incidence had previously been reported in 2002 with 597 laboratory-confirmed cases. 

The aim of this rapid communication is to increase awareness of an upcoming SINV epidemic in 2022. The high SINV incidence in 2021 may precede a larger epidemic.

## Outbreak description

**Figure 1:**
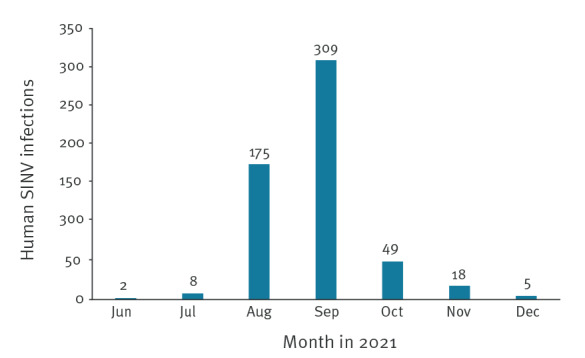
Reported cases of Sindbis virus infection by month, Finland, 2021 (n = 566)

We retrieved laboratory-confirmed cases data for the years 2002 and 2021 from the NIDR. The data included date of specimen collection and place of residence at the time of diagnosis at hospital district level. Most of the SINV infections in 2021 were diagnosed in September (n = 309) and August (n = 175) (Figure 1). A considerably smaller number of cases were reported in October (n = 49), November (n = 18), July (n = 8), December (n = 5) and June (n = 2).

SINV: Sindbis virus.

**Figure 2:**
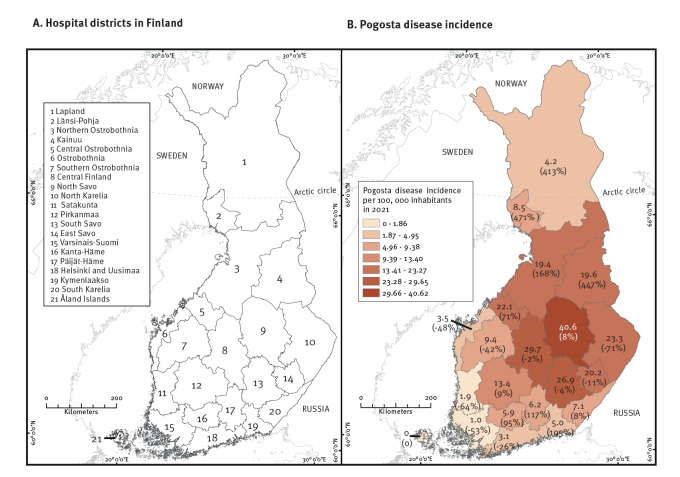
Pogosta disease incidence per 100,000 inhabitants, by the hospital district, Finland, 2021 (n = 566)

 Incidence rates of Pogosta disease in 2021 ranged in the different hospital districts from 0 (Åland Islands) to 40.6 (North Savo) per 100,000 residents (Figure 2A). The hospital districts with the highest incidences were located in central, eastern and western Finland (Figure 2B). In contrast, the lowest incidences were found in hospital districts in Lapland, along the southern coast and along the western coast southwards from Central Ostrobothnia.

Change in incidence from the earlier epidemic year 2002 to 2021 is shown in parentheses (as percentage changes for higher incidences in 2021 and negative percentage changes for lower incidences).

Although some hospital districts reported large numbers of cases in both outbreaks (2002 and 2021), there were also clear differences (Figure 2B). The SINV incidences in western and southern coastal hospital districts were lower in 2021 than in 2002 (by a range of 26–64%). The largest change in SINV infection incidence occurred in North Karelia (−71%), although SINV incidence rate in this hospital district remained high (23.3 per 100,000 inhabitants). Finally, SINV incidences were remarkably higher in several hospital districts in which not many SINV infection cases have typically been reported: Lapland, Länsi-Pohja, Kainuu, Central Ostrobothnia, North Ostrobothnia, Päijät–Häme, Kanta–Häme and Kymenlaakso (range: 71–471%) (Figure 2B).

## Discussion

The 2021 SINV outbreak was, to the best of our knowledge, the largest mosquito-borne viral disease outbreak in Europe that year. In comparison, West Nile virus caused 159 documented cases in the European Union and European Economic Area during the same mosquito season [9]. Pogosta disease cases peak in Finland typically between August and September, so it remains to be seen if there will be high case numbers this year. So far, the climatic conditions in 2022 have been favourable for mosquito abundance as in late winter, snow coverage was thick, generating a large amount of melting waters for the early season mosquitoes to breed in [10]. The grouse populations have also been increasing in Finland, providing more amplifying hosts for the SINV [11]. Also, larger numbers of mosquitoes have so far been observed, even though the mosquito season is still ongoing. All these factors create favourable prerequisites for enhanced SINV transmission in 2022.

The NIDR data for Pogosta disease cases allowed a comparison between different years by hospital district in Finland. However, the data were based on place of residence of the patient, which may not have reflected the location where the patient was infected. Despite this potential limitation, the available data suggested an expansion of the geographical range of Pogosta disease in 2021. We observed a considerably higher incidence in eight hospital districts compared with the previous outbreak in 2002. The positive change in incidence was highest in northern parts of the country and in four hospital districts with previously low incidence in southern Finland. The spatial pattern of highest incidences remained similar, which is in line with the spatial modelling results of the current SINV infection risk in Finland [12]. The typical peak in cases in August to September was also observed in the outbreaks of 2002 and 2021. In 2021, weather conditions were favourable for mosquito-borne transmission. The winter was snowy and the spring rainy, providing plenty of breeding grounds for the first mosquito generations. Furthermore, the summer was exceptionally warm, with record-high monthly mean air temperatures in June in southern and central parts of the country [13]. July was also warmer than the average [14]. Such conditions and high densities of the SINV mosquito vector and grouse population [12,15] have previously been associated with increased risk for Pogosta disease. Also, outdoor activities, which were especially popular in 2021 because of the coronavirus disease pandemic have been associated with the Pogosta disease risk [16].

The reasons for the observed geographical shift in the high incidence areas between the latest two Pogosta disease outbreaks are currently not known. The remaining immunity in the human population after the 2002 outbreak may have lowered the number of cases in the traditional endemic areas in 2021 but does not explain the spread to new areas. Further information would be needed on the factors affecting SINV emergence, including a possible effect of virus strain variation [17]. The longevity of the protective immunity after SINV infection also requires further investigation but re-infections have not been reported and for another mosquito-borne alphavirus, chikungunya virus, long-lasting protective immunity has been shown [18,19]. 

Although the large outbreaks in 2002 and 2021 have probably increased awareness of the disease since then, it is likely that Pogosta disease is underdiagnosed in Finland, especially in areas where the disease is not common. The correct diagnosis of SINV patients is important because of the potential burden of persistent joint symptoms that can last for years. These symptoms have been reported in Finland in 24.5%, and more recently in Sweden up to 39%, of the diagnosed patients [20,21].

## Conclusion

Sindbis virus caused the largest outbreak of mosquito-borne viral disease in the EU in 2021, with 566 diagnosed cases in a single country. The factors contributing and enabling SINV outbreaks are currently poorly understood. Raising public awareness of the disease and the ways of preventing mosquito bites would be important, especially in current high incidence and predicted risk areas in central, eastern and western Finland. One year after the 2002 outbreak year, elevated numbers of cases were reported in Finland and, therefore, we consider SINV transmission potential increased also for the 2022 mosquito season. The observed regional shift of reported cases to new areas poses challenges for the recognition of the disease and highlights the need for using virus-specific diagnostic testing of febrile patients with compatible symptoms.

## References

[1] Brummer-Korvenkontio M Vapalahti O Kuusisto P Saikku P Manni T Koskela P Epidemiology of Sindbis virus infections in Finland 1981-96: possible factors explaining a peculiar disease pattern. Epidemiol Infect. 2002;129(2):335-45. 10.1017/S0950268802007409 12403109 PMC2869892

[2] Kurkela S Manni T Myllynen J Vaheri A Vapalahti O. Clinical and laboratory manifestations of Sindbis virus infection: prospective study, Finland, 2002-2003. J Infect Dis. 2005;191(11):1820-9. 10.1086/430007 15871114

[3] Laine M Luukkainen R Jalava J Ilonen J Kuusistö P Toivanen A. Prolonged arthritis associated with sindbis-related (Pogosta) virus infection. Rheumatology (Oxford). 2000;39(11):1272-4. 10.1093/rheumatology/39.11.1272 11085809

[4] Adouchief S Smura T Sane J Vapalahti O Kurkela S. Sindbis virus as a human pathogen-epidemiology, clinical picture and pathogenesis. Rev Med Virol. 2016;26(4):221-41. 10.1002/rmv.1876 26990827

[5] Kurkela S Rätti O Huhtamo E Uzcátegui NY Nuorti JP Laakkonen J Sindbis virus infection in resident birds, migratory birds, and humans, Finland. Emerg Infect Dis. 2008 a;14(1):41-7. 10.3201/eid1401.070510 18258075 PMC2600146

[6] Manni T Kurkela S Vaheri A Vapalahti O. Diagnostics of Pogosta disease: antigenic properties and evaluation of Sindbis virus IgM and IgG enzyme immunoassays. Vector Borne Zoonotic Dis. 2008;8(3):303-11. 10.1089/vbz.2007.0623 18380591

[7] Kurkela S Manni T Vaheri A Vapalahti O. Causative agent of Pogosta disease isolated from blood and skin lesions. Emerg Infect Dis. 2004;10(5):889-94. 10.3201/eid1005.030689 15200824 PMC3323234

[8] Finnish Institute for Health and Welfare (THL). Tartuntatautirekisterin tilastotietokanta. [Statistical database of National Infectious Disease Registry]. Helsinki: THL. [Accessed 22 Jun 2022]. Finnish. Available from: https://sampo.thl.fi/pivot/prod/fi/ttr/shp/fact_shp?row=area-12260&column=time-12059&filter=reportgroup-12074

[9] European Centre for Disease Prevention and Control (ECDC). West Nile virus infections, 2021 transmission season. Stockholm: ECDC; 2022. Available from: https://www.ecdc.europa.eu/en/publications-data/west-nile-virus-infections-2021-transmission-season

[10] Finnish Meteorological Institute (FMI). Record-high precipitation in Finland in February. Helsinki: FMI; 2022. Available from: https://en.ilmatieteenlaitos.fi/press-release/2h3QJL1lTT0XS0ngb9BRl8

[11] Natural Resources Institute of Finland Luke. Riistakolmiolaskennat kesä 2021: metsäkanalintujen pesintä onnistui hyvin. [Luke: Game triangulation calculations summer 2021: nesting of grouse was successful]. Helsinki: Finnish Wildlife Agency; 2021. Finnish. Available from: https://riista.fi/luke-riistakolmiolaskennat-kesa-2021-metsakanalintujen-pesinta-onnistui-hyvin

[12] Uusitalo R Siljander M Culverwell CL Hendrickx G Lindén A Dub T Predicting spatial patterns of Sindbis virus (SINV) infection risk in Finland using vector, host and environmental data. Int J Environ Res Public Health. 2021;18(13):7064. 10.3390/ijerph18137064 34281003 PMC8296873

[13] Finnish Meteorological Institute (FMI). May brought hot days and heavy rain. Helsinki: FMI; 2021. Available from: https://en.ilmatieteenlaitos.fi/press-release/48Q6w0KSao3EUxPywKMaHo

[14] Finnish meteorological Institute (FMI). Normal average temperature in 2021 ‒ summer was warm. Helsinki: FMI; 2022. Available from: https://en.ilmatieteenlaitos.fi/press-release/14Fa6a8VkiZ9cHCityfxPF

[15] Jalava K Sane J Ollgren J Ruuhela R Rätti O Kurkela S Climatic, ecological and socioeconomic factors as predictors of Sindbis virus infections in Finland. Epidemiol Infect. 2013;141(9):1857-66. 10.1017/S095026881200249X 23158410 PMC9155282

[16] Sane J Guedes S Ollgren J Kurkela S Klemets P Vapalahti O Epidemic sindbis virus infection in Finland: a population-based case-control study of risk factors. J Infect Dis. 2011;204(3):459-66. 10.1093/infdis/jir267 21742846

[17] Korhonen EM Suvanto MT Uusitalo R Faolotto G Smura T Sane J Sindbis virus strains of divergent origin isolated from humans and mosquitoes during a recent outbreak in Finland. Vector Borne Zoonotic Dis. 2020;20(11):843-9. 10.1089/vbz.2019.2562 32898458 PMC7699012

[18] Auerswald H Boussioux C In S Mao S Ong S Huy R Broad and long-lasting immune protection against various Chikungunya genotypes demonstrated by participants in a cross-sectional study in a Cambodian rural community. Emerg Microbes Infect. 2018;7(1):13. 10.1038/s41426-017-0010-0 29410416 PMC5837154

[19] Nitatpattana N Kanjanopas K Yoksan S Satimai W Vongba N Langdatsuwan S Long-term persistence of Chikungunya virus neutralizing antibodies in human populations of North Eastern Thailand. Virol J. 2014;11(11):183. 10.1186/1743-422X-11-183 25330992 PMC4283153

[20] Gylfe Å Ribers Å Forsman O Bucht G Alenius GM Wållberg-Jonsson S Mosquitoborne Sindbis virus infection and long-term illness. Emerg Infect Dis. 2018;24(6):1141-2. 10.3201/eid2406.170892 29781426 PMC6004841

[21] Kurkela S Helve T Vaheri A Vapalahti O. Arthritis and arthralgia three years after Sindbis virus infection: clinical follow-up of a cohort of 49 patients. Scand J Infect Dis. 2008 b;40(2):167-73. 10.1080/00365540701586996 17852949

