# Erratum for Euro Surveill. 2022;27(31)

**DOI:** 10.2807/1560-7917.ES.2022.27.35.220901e

**Published:** 2022-09-01

**Authors:** Eurosurveillance editorial team

**Affiliations:** 1 European Centre for Disease Prevention and Control (ECDC), Stockholm, Sweden

In the article ‘Sindbis virus outbreak and evidence for geographical expansion in Finland, 2021’ by Suvanto et al., published on 04 August 2022, the funding statement was erroneously left out in the originally published version. This was corrected on 1 September 2022. We apologise for any inconvenience this may have caused.

